# Robot and working tube-assisted invasion-controlled surgery for spinal metastases

**DOI:** 10.3389/fsurg.2023.1041562

**Published:** 2023-02-24

**Authors:** Shangbin Zhou, Bo Li, Pengru Wang, Meiling Xu, Jian Zhao, Shujie Duan, Zhipeng Zhu, Wei Xu, Jianru Xiao

**Affiliations:** ^1^ Department of Orthorpedic Oncology, Changzheng Hospital, Naval Military Medical University, Shanghai, China; ^2^ Naval Medical Center, Naval Military Medical University, Shanghai, China; ^3^ Department of Radiology, Changzheng Hospital, Naval Military Medical University, Shanghai, China

**Keywords:** spinal metastases, invasion-controlled surgery, minimally invasive, robot, working tube

## Abstract

**Objective:**

This study aims to highlight the use of robots in surgery and that of tube-assisted minimally invasive surgery for spinal metastases, as well as elaborate on the concept of invasion-controlled surgery (ICS).

**Summary of background:**

Many patients with spinal metastasis cancer cannot afford serious complications when undergoing traditional open surgery because of their poor physical condition. Robots and minimally invasive technology have been introduced into the field of spine surgery and they have shown significant advantages.

**Methods:**

Six patients who underwent robot and working tube-assisted ICS for spinal metastases. Relevant demographic, medical, surgical, and postoperative data were collected from medical records and analyzed.

**Results:**

Mean operative time was 3.8 h and the mean length of the surgical incision was 4.9 cm. The mean estimated blood loss was 400 ml. The mean bedtime and hospital length of stay were 3.2 days and 6.5 days, respectively. No obvious complications were reported during treatment. The mean accuracy of screw placement was 98%. The mean time for further system treatment after surgery was 5.8 days. All patients experienced significant pain relief. The mean preoperative visual analog scale (VAS) was 7.83 points. The mean VAS at 1 day, 1 week, and 1 month after surgery were 2.83, 1.83, and 1.17 points, respectively. Frankel grade was improved in five of six patients. One patient preoperatively with Frankel grade D was the same postoperatively.

**Conclusion:**

The concept of ICS is suitable for patients with spinal metastases. Robot and working tube-assisted ICS for spinal metastases is one of the safest and most effective treatment methods.

## Introduction

According to the GLOBOCAN 2020 estimates of cancer incidence and mortality produced by the International Agency for Research on Cancer, an estimated 19.3 million new cancer cases were reported worldwide (1). Approximately 70% of cancer patients have spinal metastases (2). In recent years, the incidence of spine metastases is increasing due to the aging population and improved targeted therapy (3). Roughly one-third of patients with spinal metastases develop symptoms, including intractable pain, neurological dysfunction, and/or spinal instability (4). Surgical treatment is an effective means to solve these issues (5, 6) and has been evolving towards approaches that are both precise and minimally invasive (7).

Nonetheless, numerous patients with spinal metastasis cancer cannot afford serious complications that might result from undergoing traditional open surgery due to their poor physical condition (8) or relatively short survival time. Moreover, a comprehensive review report by Gelalis et al. (9) stated that pedicle screw misplacement rates were 0.1% to 31% using the freehand technique (10). A misplaced pedicle screw can result in complications including dural tearing, neural damage, and vascular or visceral injury. The rate of complications in relation to pedicle screw misplacement increased to 54% (11–14). Traditional open surgery complications and pedicle screw misplacement will result in a slower recovery time or a further decline in life quality.

Robots and minimally invasive techniques have been introduced into the field of spine surgery and they have demonstrated outstanding advantages. Numerous studies suggested that robot-assisted spine surgery offers screw placement accuracy, efficiency, as well as superior safety, when compared to the freehand technique (15). Robot-assisted technology could significantly reduce unnecessary tissue separation exposure and cause lower blood loss (16). In the meantime, the intra-operative radiation dose would also be reduced (15). Furthermore, with the application of targeted drugs and immune checkpoint inhibitors, the systemic control of many patients with malignant tumors is optimistic. Due to the lengthy healing period and potential complications associated with open surgical incisions, it would be inadvisable to implement the aforementioned treatment methods immediately.

In this study, we proposed the concept of invasion-controlled surgery (ICS) for spinal metastasis. It is a minimally invasive surgery that aims to enhance, in a short time, the quality of life of patients who have spinal metastasis by performing a well-controlled operation. Based on this surgical concept, we implemented a new surgery on six patients: robot and working tube-assisted ICS of spinal metastases. This article describes the indications, characteristics, and patient benefits of this surgical procedure.

## Material and methods

### Patients' selection

**Figure 1:**
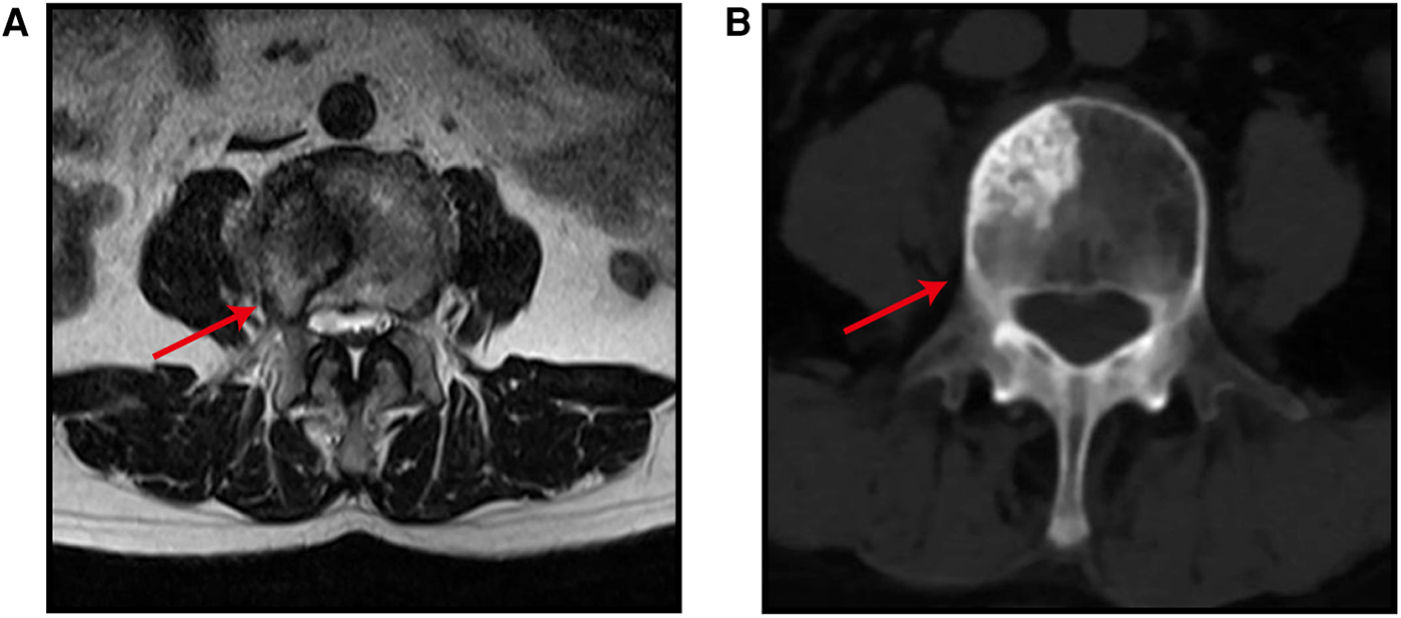
MRI image (A) and CT image (B) show metastatic tumor involve unilateral articular joints and cause nerve root involvement and mild epidural compression. The red arrow points to the location of the lesion.

Six patients were diagnosed with malignant tumors and spinal metastases between May and August of 2021. There were five men and one woman with an average age of 59 years (range 50–71 years) and an average Karnofsky Performance Scale (KPS) score of 75 (range 70–80). All patients had symptoms of mechanical pain, nerve root, or/and spinal cord compression. Each patient signed informed consent and underwent robot and working tube-assisted ICS of spinal metastases. The robot was manufactured by Shanghai Jiao Information Technology Development Co., Ltd., and the catheter was manufactured by Medtronic, Inc. All patients had a limited life expectancy, with a mean revised Tokuhashi score of 8.8 (range 7–11). Table 1 provides a summary of the preceding key statistics. Before surgery, the patients underwent anteroposterior and lateral radiographs, computed tomography (CT), and magnetic resonance imaging (MRI) examination of the segment with the lesion and two to three adjacent segments. The average spine instability neoplastic score (SINS) was 10.2 points (range 8–13 points) according to the CT image. MRI demonstrated lesions were all located in the lower thoracic spine or lumbar spine. The lesions involved unilateral articular joints and led to unilateral nerve root or/and spinal cord compression. CT and MRI images are depicted in Figure 1. The responsible lesion segment involves a part of the side of the vertebral body and yet does not exceed the midline of the vertebral body. The patients with spinal metastasis from a malignant tumor required surgical intervention, but their physical condition was generally poor, and the minimally invasive surgical treatment plan was determined through multidisciplinary consultation.

**Table 1:** Summary of primary demographic data in 6 patients who underwent ICS for spinal metastasis.

NO.	Age (yrs), Sex	Type of Cancer	KPS	Responsible lesion level*	Revised Tokuhashi score	SINS	ESCCs^#^	History of primary tumor treatment	History of systemic therapy	History of treatment for spinal metastases	Frankel score Preop.	VAS Preop.
1	61, M	Lung cancer	70	L4	9	9	1b	Lung cancer resection	Pemetrexed 900 mg d1+ Nedaplatin 120 mg d1, q3 w, ivgtt. Pemetrexed 900 mg + Carboplatin 600 mg d1, q3 w + Camrelizumab 200 mg d1, q3 w, ivgtt.	None	C	9
2	50, W	Lung cancer	80	T12	9	8	2	None	None	None	C	7
3	66, M	Prostate cancer	70	L4	11	10	1a	None	Bicalutamide Tablets 50mg qd, *p*.o. Leuprorelin Acetate Microspheres for Injection q4 w ih. Zoledronic acid Injectiononce, q4 w, ivgtt.	None	C	8
4	53, M	Lung cancer	70	L4	7	9	1a	Lung cancer resection	Gefitinib Tablets 250 mg, qd, *p*.o.	42Gy/14f	D	6
5	71, M	Prostate cancer	80	L2	10	13	2	Trans Urethral Resection Prostate (TURB)	Abiraterone Acetate Tablet 1000 mg qd, *p*.o. Leuprorelin Acetate Microspheres for Injection q4 w ih. Zoledronic acid Injectiononce, q4 w, ivgtt.	None	C	8
6	52, M	Lung cancer	80	T11	7	12	2	None	Pemetrexed 700 mg + Carboplatin 400 mg d1, q3 w, ivgtt.	None	C	9

* Responsible lesion level: The level of the lesion that causes the symptoms.

^#^ ESCCs: Epidural Spinal Cord Compression Scale.

### Surgical techniques and tools

**Figure 2.**
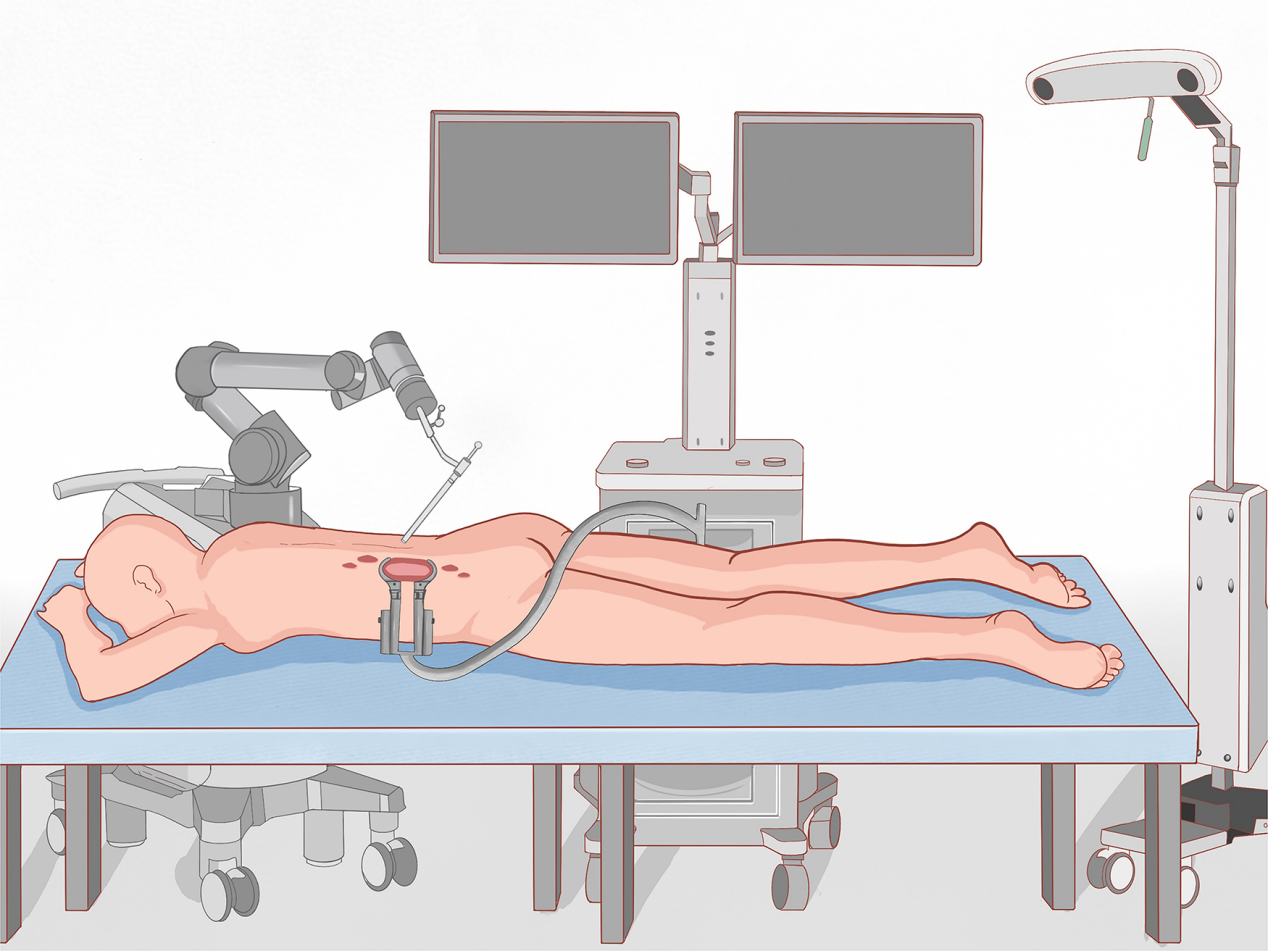
Scene diagram of the surgical process.

**Figure 3.**
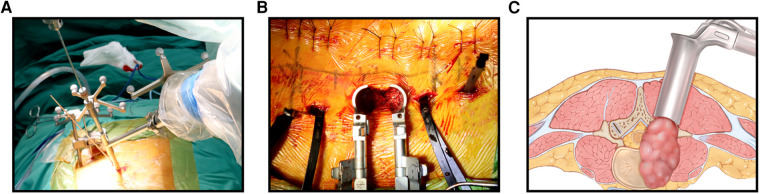
(A): Robot-assisted positioned the skin projection of the pedicle screw entry point. (B): Complete decompression and resection under the working tube. (C): Schematic diagram of the working tube and the extent of the lesion.

Before surgery, valuable spine imaging was obtained, including anteroposterior and lateral radiographs, and a thin-slice (1-mm) CT scan of the responsible lesion segment and the upper and lower two or three adjacent vertebral spine levels. Based on these images, we built a virtual three-dimensional (3D) model of the spine on the robot host software and determined the appropriate length, diameter, direction, and insertion angle of the pedicle screw on this model. During surgery, the patient was positioned prone on a radiolucent operating table, allowing for anteroposterior and lateral fluoroscopic control with x-rays (Figure 2). Furthermore, the robot positioned the skin projection of the pedicle screw entry point one by one and created a small incision of approximately 1 cm on the skin (Figure 3A). With the assistance of the prepared robot, the placement of pedicle screws along the predetermined trajectory was performed individually. The screws we employed were bone cement injectable canulated pedicle screws (CICPS, from Shanghai Sanyou Medical Co., Ltd). After the x-ray fluoroscopy displayed that the position and direction of the screw were appropriate, we inserted the bone cement into the CICPS. The number of x-ray fluoroscopy during surgery was counted (Table 2). We also employed the robot to accurately locate the skin projection of the responsible lesion segment and make a 3 cm-5 cm incision at the location. The thoracolumbar fascia was exposed, and its incision allowed for a Wiltse muscle-splitting technique-compliant gentle dissection between the muscles (17). Moreover, the Wiltse approach allowed direct access to the unilateral articular joints. We consequently maintained and expanded the space between the muscles by introducing an expandable working tube **(**Figure 3B,C). The diameter was 22 mm when not expanded. When expanded, the maximum width of the upper part was 50–55 mm, and the maximum width of the lower part was 80–100 mm. The working tube was widened and moved moderately as required to visualize the altered anatomy. Unilateral lamina, facet joints, and pedicle could be removed to complete dorsal decompression as well as nerve root release under the expandable working tube **(**Figure 3C**)**. The part of the vertebral body involved by the lesion (not more than the midline of the vertebral body), was removed to complete the ventral side stress reliever. After the resection, the stability of the anterior column of the vertebral body could be compromised. In this case, namely case 3, we placed a proper amount of bone cement in the missing vertebral body position *via* the expandable working tube. Accordingly, the scope of the lesion that could be excised under the expandable working tube was 1–6 or 7–12 areas of the Weinstein-Boriani-Biagini spine surgical staging (WBB staging), and WBB staging areas 2–3 or 10–11 are the most convenient for operations. After the procedure, the longitudinal connecting rod was inserted, the nut was tightened, and a drainage tube was placed at the excision site. All patients underwent further systematic treatment, such as targeted therapy, immunotherapy, or chemotherapy, under conditions permitted by their bodies following surgery, and the time spent receiving treatment was tallied (Table 2).

**Table 2:** Postoperative data of primary demography in 6 patients.

NO.	Incision length (cm)^&^	Estimated blood loss (ml)	Operation time (h)	The number of intraoperative fluoroscopy	Bedtime (d)	Length of hospital stay (d)	The accuracy of screw placement (%)	Driver gene	Further systematic treatment	Time to receive further systematic treatment (d)	Frankel score Postop.	VAS One day Postop.	VAS One week Postop.	VAS One month Postop.
1	5	600	4	4	2	7	100	None	Paclitaxel (Albumin Bound) 400 mg + Carboplatin 600mg + Camrelizumab 200 mg d1, q3 w, ivgtt. + Bevacizumab 400 mg d1, q3 w, ivgtt. Denosumab 120 mg, q4 w, ih.	10	E	3	2	2
2	3.5	200	2	3	3	6	87.5	EGFR (21L858R)	Gefitinib Tablets 250 mg, qd, *p*.o. Denosumab 120 mg, q4 w, ih.	10	E	2	2	1
3	5	500	4	5	3	6	100	None	Bicalutamide Tablets 50 mg qd, *p*.o. Leuprorelin Acetate Microspheres for Injection, q4 w ih. Denosumab 120 mg, q4 w, ih.	0	E	3	1	1
4	5	400	4.5	3	4	5	100	EGFR (T790M)	Aclitaxel (Albumin Bound) 400 mg + evacizumab 400 mg d1, q3 w, ivgtt. Docetaxel 120 mg, ivgtt. Osimertinib Mesylate Tablets 80 mg qd, *p*.o. Denosumab 120 mg, q4 w, ih.	7	D	3	2	2
5	5	300	5	4	3	8	100	None	Abiraterone Acetate Tablet 1000 mg qd, *p*.o. Leuprorelin Acetate Microspheres for Injection, q4 w ih. Denosumab 120 mg, q4 w, ih.	0	E	3	1	0
6	6	400	3.5	3	4	7	100	EGFR (21L858R)	Osimertinib Mesylate Tablets 80 mg qd, *p*.o. Denosumab 120 mg, q4 w, ih.	8	D	3	3	1

^&^ Incision length: The length of the incision where the working tube was placed.

## Results

During the patient's hospitalization, data on operation time, incision length, operation time, estimated blood loss during the operation, bedtime, hospital stay, and complications were collected. Visual analog scales (VAS) and the Frankel grade classification were employed to, respectively, measure pain and neurological deficit before surgery and at discharge. The accuracy of screw placement was evaluated by CT imaging according to a Gertzbein and Robbins classification system (18): Grade A, in the pedicle; Grade B, perforation <2 mm; Grade C, perforation >2 mm but < 4 mm; Grade D, perforations > 4 mm but <6 mm; and Grade E, perforations >6 mm. Grade A and Grade B were regarded as accurate nail placement (19). This data is displayed in Table 2.

### Operative data

The mean operative time was 3.8 h (range 2–5 h) and the mean length of the surgical incision of the decompression tube was 4.9 cm (range 3.5 cm–6 cm). The mean estimated blood loss was 400 ml (range 200–600 ml), and no patient needed a blood transfusion during or after the operation. The mean bedtime and length of hospital stay were 3.2 days (range 2–4 days) and 6.5 days (range 5–8 days), respectively.

### Complications

There were no reported complications during treatment. All patients underwent x-ray and CT scans after surgery and the mean accuracy of the robot-assisted screw placement was 98% (range 87.5%–100%). During the duration of the study, no instrument failures were reported. The mean time to receive further systematic treatment was 7.67 days (range 0–21 days). The mean follow-up time was 5 months (range 4–7 months), and no patients were lost during follow-up. During the follow-up, it was determined that no patients had wounds that did not heal properly or other adverse surgical complications.

### Neurological course

**Figure 4.**
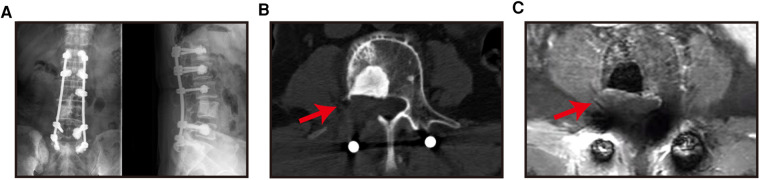
X-ray (A), CT images (B) and MRI images (C) of patients were reviewed 6 months after surgery.

Four of the six patients (66.66%) improved by two Frankel grades (from C to E), and these four patients were able to walk independently without pain during hospitalization. One patient (16.66%) improved by one Frankel grade (from C to D grade). One patient (16.66%, Case 4), who had a Frankel grade D before the operation was still grade D after the operation, and yet the patient claimed the pain was significantly reduced. No patients experienced pain or spinal cord compression at the treatment level again during the follow-up period (Figure 4).

### Pain alleviation

**Figure 5.**
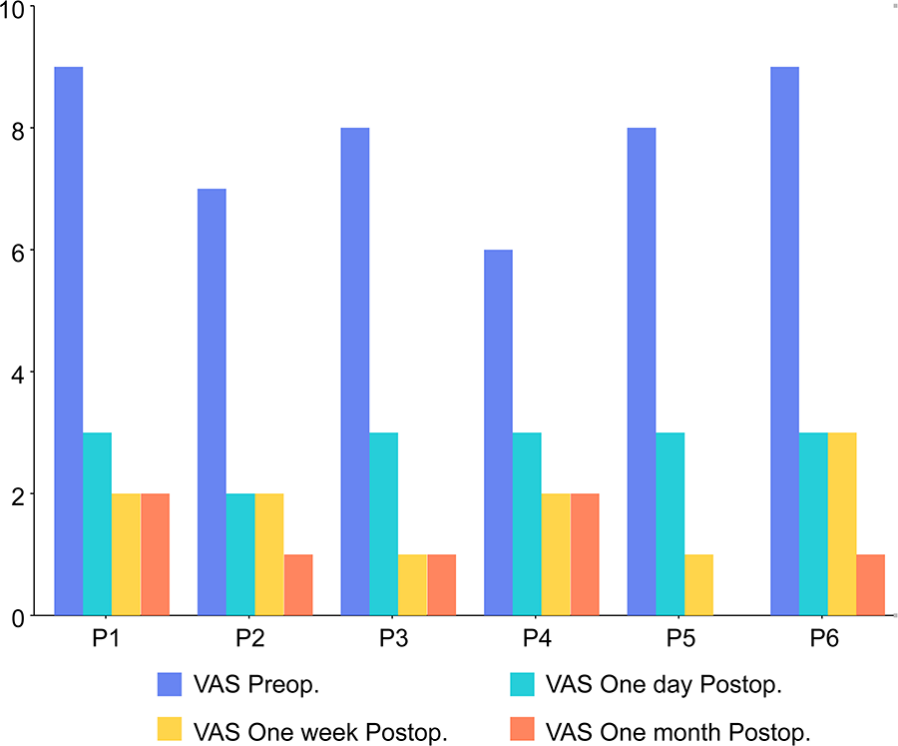
The X-ray (A), CT image (b) and MRI image (C) of the patient at 6 months after the operation showed spine no local progression (the red arrow is the decompression area, and item with high density at the vertebral body in B is intraoperative bone cement).

All patients' pain were significantly reduced. (Figure 5) as they all experienced massive pain relief. The mean preoperative VAS was 7.83 points. The mean VAS at 1 day, 1 week, and 1 month after surgery were 2.83, 1.83, and 1.17 points, respectively, meaning a decrease of more than 5 points. During the period of follow-up, none of the patients experienced a recurrence of pain.

## Discussion

In patients with malignant tumors, spinal metastasis is more prevalent. It may cause severe symptoms and serious damage to the quality of life. Once neurological signs are present, there is level I evidence that direct decompressive surgery is far superior to radiotherapy alone in patients with spinal metastasis (20). The revised Tokuhashi score (21) asserts that the majority of patients with lung cancer have a survival period of fewer than 6 months. Nonetheless, during the last decades, clinicians have witnessed remarkable developments in molecular targeted therapy, particularly the second development of immune checkpoint inhibitors that have significantly enhanced the prognosis of lung cancer patients (22). Consequently, we can perform palliative surgery on lung cancer patients with neurological signs. The surgical treatment is aimed at palliative care (23)—to relieve pain, repair and protect nerve function, and correct spinal instability, as well as strengthen the quality of life of patients. Notwithstanding, conventional open surgery may cause significant bleeding during the operation and there are numerous risks involved, including excessive muscle trauma, and nerve and blood vessel damage, which may cause major complications after surgery. Additionally, the risks of conventional open surgery will be more prominent for patients who are elderly, with underlying diseases, poor nutritional status as well as for those who have previously received chemotherapy, radiotherapy, or glucocorticoid therapy. Hence, the proposed concept of ICS for spinal metastases benefits patients in several ways.

The six patients with spinal metastases who were included in this study all had nerve root pain, spinal instability, or neurological dysfunction. Vertebroplasty could not effectively solve these problems. ICS was the most reliable method of surgery to rebuild spine stability and remove the responsible lesions, and thus decompress the spinal cord and nerve roots.

The robot-assisted technique is a superior method to place pedicle screws in the spine, due to its higher accuracy, safety, and the feasibility rates of the procedure. It could benefit from the following characteristics. Firstly, the data of preoperative robot planning has the advantages of intuitiveness, accuracy, and repeatability. Secondly, the intraoperative fluoroscopy image is synchronized with the preoperative CT image during the operation, which is one of the reasons why high accuracy can be achieved. Additionally, the robot can also avoid most human distinctions and fatigue-related errors, such as a surgeon's trembling hands or poor coordination. These features allow the robot to have an active role in screw placement precision, operation time, and radiation exposure.

On the other hand, we analyzed the characteristics of the patients' lesions, which predominantly affected unilateral facet joints, resulting in unilateral nerve root or spinal cord compression. The operation under the small incision and expandable working tube was able to achieve the purpose of resectioning the lesion and relieving the compression with minimal invasion, which also incorporated the ICS concept for spinal metastasis. Consequently, the mean surgical incision was 4.9 cm, the mean intraoperative blood loss was 400 ml, the postoperative neurological function of the patient improved, and the pain was relieved. These and the absence of obvious complications confirm the efficacy of this minimally invasive technique.

Patients with spinal metastases should receive additional systematic treatment as soon as possible after surgery, that is, they should transition from short-term palliative surgery to long-term tumor control. Nonetheless, the prerequisites are that a patient has no obvious postoperative complications, the surgical wound heals and the systemic condition recovers as soon as possible. These are the exact objectives pursued by ICS.

Nevertheless, the implementation of such surgery also has some constraints. For instance, the popularity of spinal robots is not significant, the range of decompression under the working tube cannot be overly large, and the operation of decompression under the working tube requires an experienced spinal surgeon to perform the operation.

## Conclusions

The concept of invasion-controlled surgery is extremely suitable on the condition that patients with malignant spinal metastases and limited life expectancy require surgical treatment. Moreover, the six cases of surgery in this article were specific cases of ICS for spinal metastasis, which further indicates that it is a safe and effective method of treatment that can lessen complications, promote rapid recovery of patients after surgery, and enable patients to enter more quickly the next step of treatment.

## Data Availability

The original contributions presented in the study are included in the article/Supplementary Material, further inquiries can be directed to the corresponding author/s.
